# Multiple Mucous Cysts on the Hands of a Patient With Systemic Sclerosis Treated With Collagen Sheets for Scar Healing

**DOI:** 10.1111/1346-8138.17763

**Published:** 2025-04-28

**Authors:** Takano Hobo, Hiroshi Kato, Shinji Kano, Yukiko Yasui, Maki Yoshimitsu, Motoki Nakamura, Akimichi Morita

**Affiliations:** ^1^ Department of Geriatric and Environmental Dermatology Nagoya City University Graduate School of Medical Sciences Nagoya Japan

**Keywords:** collagen sheets, mucin, multiple mucous cysts, surgery, systemic sclerosis, wound healing

Dear Editor,

A 20‐year‐old man was referred to our clinic after developing multiple blisters on his fingers 2 years ago. He was diagnosed with systemic sclerosis 7 years ago and treated by a local physician. At his initial visit, he presented with multiple tense blisters measuring 1–2 cm in diameter on his fingers, accompanied by sclerosis (Figure 1a,b). Laboratory tests revealed positive anti‐Scl‐70 antibodies, while pemphigoid antibodies were negative.

**FIGURE 1:**
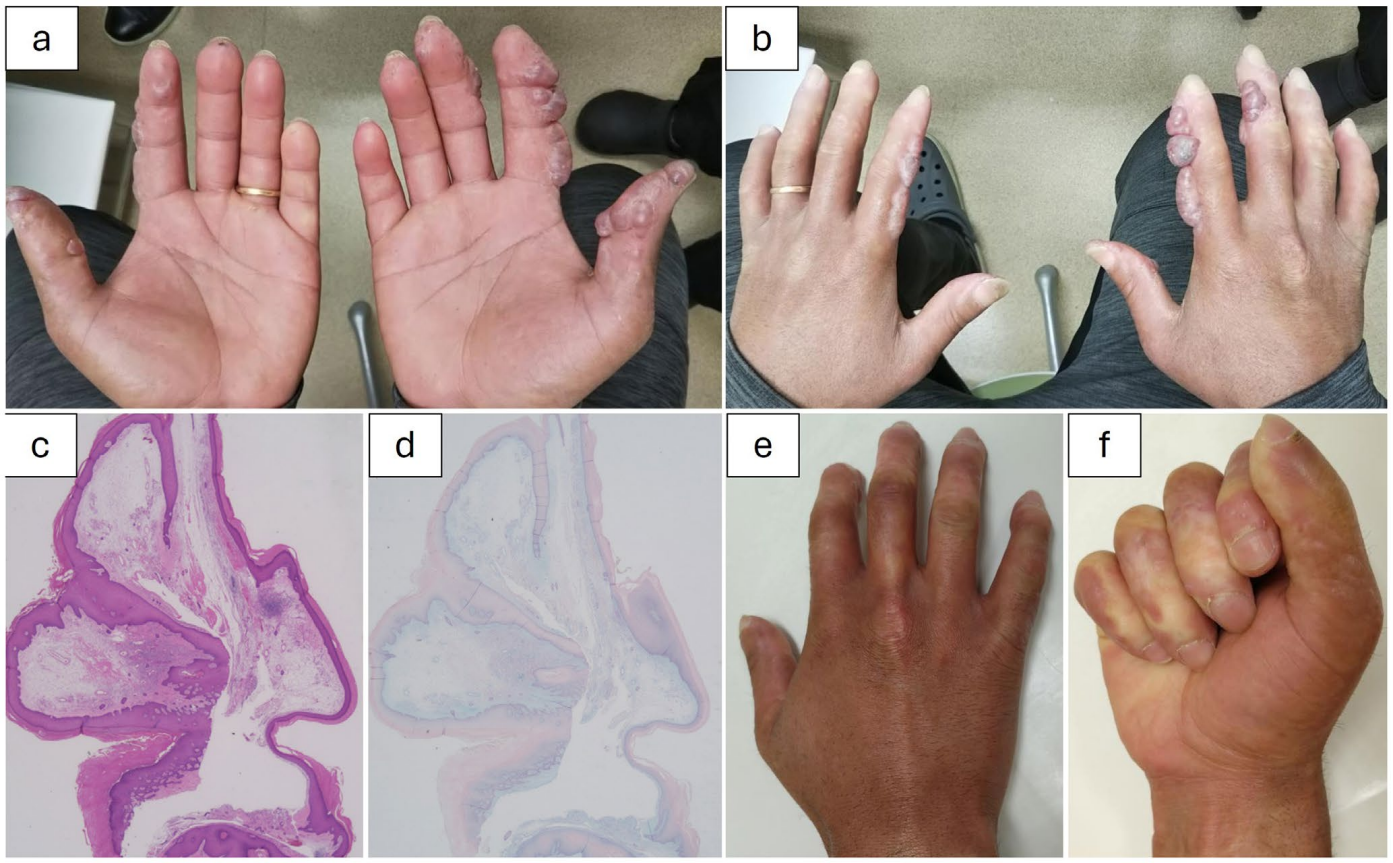
(a) Clinical photograph of the palm displaying blisters. (b) Clinical photograph showing multiple blisters on the dorsal side of the right first, second, and third fingers, as well as the left first and second fingers. (c) Hematoxylin and eosin (HE) staining of the excised specimen, revealing areas lacking defined structure and exhibiting edema in the interstitial tissue of the subcutaneous layer. (×20) (d) Immunostaining of the excised specimen with Alcian blue (pH 2.5), demonstrating positive staining in the interstitial tissue. (×20) (e) Clinical photograph of the dorsal hand 1 year postoperatively, demonstrating no recurrence of lesions. (f) Clinical photograph of the palm 1 year postoperatively, showing no contracture and preserved grasping function.

For diagnosis, a blister was completely excised under local anesthesia, which contained a viscous fluid. Histopathological examination revealed a cystic lesion with an indistinct wall structure, and Alcian blue staining demonstrated mucin deposition in the interstitial tissue, confirming mucinous cysts (Figure 1c,d). Following excisional biopsy, a collagen sheet was applied to promote scar healing. As no recurrence was observed at the biopsy site, all other lesions were excised and allowed to heal with collagen sheets. No recurrence was observed in the past year (Figure 1e,f).

Mucous cysts are generally classified into two types: the ganglion type, which arises from the distal interphalangeal joint, and the myxomatous type, which results from skin degeneration. The myxomatous type is caused by excessive hyaluronic acid production due to dermal fibroblast transformation into myxoma cells following mechanical stimulation. In systemic sclerosis, mucin deposition occurs due to excessive extracellular matrix protein production by activated fibroblasts, resulting in mucinous edema [1]. Similar to previous case reports [2], the lesions in this case were primarily concentrated in easily irritated areas rather than regions with severe skin sclerosis. This suggests that the cysts were caused by irritation combined with the existing scleroderma‐type skin sclerosis.

In this case, mucin deposition in sclerotic skin associated with systemic sclerosis likely contributed to the formation of mucinous cysts with a distinctive appearance. Mucin deposition in scleroderma is driven by fibroblast stimulation via inflammatory cytokines, including IL‐1 and IL‐6 [1]. As the lesions were localized to the fingers and the patient worked as a welder, mechanical stimulation may have triggered IL‐1 and IL‐6 production. Oxidative stress plays a key role in scleroderma, and low oxygen levels promote fibrogenic activities, including fibroblast proliferation and collagen production [3]. Recent studies have suggested that hypoxia contributes to dermal mucin deposition by increasing fibroblast biosynthetic activity and hyaluronic acid production [4].

The patient had undergone multiple aspiration procedures before visiting our clinic; however, the cysts recurred rapidly. Given the large number of lesions, we opted for excision therapy. The excision of small mucous cysts is followed by suturing or skin flap reconstruction. However, due to the extent of the lesions, direct suturing or flap reconstruction was challenging, and scar healing with collagen sheets was chosen instead. Collagen sheets increase HGF production by fibroblasts and consequently reduce TGFβ, which is believed to mitigate scleroderma‐like skin changes and prevent recurrence [5].

A previous case reported that excision followed by full‐thickness skin grafting resulted in recurrence several months later [2]. Our patient has remained free of recurrence for 1 year postoperatively, suggesting that using collagen sheets for scar healing helps prevent cyst recurrence.

## Ethics Statement

We obtained written informed consent from the patient to publish his clinical details.

## Conflicts of Interest

The authors declare no conflicts of interest.
